# A Simple and Effective Colorimetric Assay for Glucose Based on MnO_2_ Nanosheets

**DOI:** 10.3390/s18082525

**Published:** 2018-08-02

**Authors:** Zhengjun Huang, Linlin Zheng, Feng Feng, Yuyuan Chen, Zhenzhen Wang, Zhen Lin, Xinhua Lin, Shaohuang Weng

**Affiliations:** Department of Pharmaceutical Analysis, School of Pharmacy, the Higher Educational Key Laboratory for Nano Biomedical Technology of Fujian Province, Fujian Medical University, Fuzhou 350122, China; jjonepo@163.com (Z.H.); linlinzheng1111@163.com (L.Z.); camelsoul@163.com (F.F.); 13655042668@163.com (Y.C.); 15980232303@163.com (Z.W.); linwenjing002@163.com (Z.L.); 13906909638@163.com (X.L.)

**Keywords:** glucose, colorimetric assay, MnO_2_ nanosheets, UV-vis, quantitative determination

## Abstract

Simple and effective methods for the detection of the level of blood glucose are closely linked to the monitoring of people’s health. In the study, MnO_2_ nanosheets with absorption range of 300 nm~500 nm and obvious yellow color were easily prepared and applied to detect glucose through their absorbance and color. The proposed method is based on the fact that a specific concentration of glucose can be quantitatively transformed into hydrogen peroxide (H_2_O_2_) under the catalytic effect of glucose oxidase. Based on the redox reaction of MnO_2_ with H_2_O_2_, yellow MnO_2_ can be converted into colorless Mn^2+^ to monitor the concentration of glucose. Under optimal conditions, a simple and effective visual assay for the sensitive and reliable detection of glucose was developed. The linear range was estimated to the range from 0 μM to 100 μM, with a detection limit of 12.8 μM. Furthermore, the proposed colorimetric assay based on MnO_2_ nanosheets can effectively detect blood glucose of clinical serum samples with accuracy and convenience.

## 1. Introduction

Glucose, the energy source of living cells and the intermediate product of metabolism, plays an important role in the natural growth of cells and organisms. For humans, blood glucose should be maintained at the suitable range to meet the needs of a healthy body. Abnormal levels of blood glucose are closely linked to some diseases such as diabetes and hypoglycemia. In recent years, the trend of younger and the increasing incidence of diabetes require some facile and accurate methods for the monitor of blood glucose. At present, numerous analytical methods, such as surface-enhanced Raman scattering [1], chemiluminescence [2], electrochemistry (electroanalysis and electrochemiluminescence) [3,4], fluorescence [5,6] and spectrophotometry [7], have been developed and applied for blood glucose determination. Among them, colorimetric assays based on spectrophotometry have attracted great attention, which has led to a significant contribution to real-time detection of glucose in a convenient and economical strategy [8,9,10]. A variety of colorimetric systems are used to accurately determine blood glucose, such as those based on paper microfluidic devices [11], metal-organic frameworks [12], nanoclusters [13], mesoporous carbon [14,15] and magnetic nanoparticles [16]. Although these methods are powerful and effective, the potential interference of complex components present in clinical specimens and high cost are still issues in visual blood glucose measurement. Therefore, it is still of great value to develop some new type colorimetric methods that are modification-free and inexpensive for blood glucose monitoring.

Hydrogen peroxide (H_2_O_2_) plays key roles as a redox signaling molecule in the regulation of various physiological processes [17,18]. The control level of H_2_O_2_ is a relatively significant indicator of several biological processes such as the immune system [19], and cancer incidence [20,21]. In addition, H_2_O_2_ can be the main intermediary reactant for immunoassays such as enzyme linked immunosorbent assay (ELISA) and chemiluminescence [22,23]. Furthermore, H_2_O_2_ is a byproduct of general oxidation reactions from the catalytic process of glucose oxidase [24], amino-acid oxidase [25], uricase and so on [26]. Thus, accurate detection of H_2_O_2_ or fabricating special sensing method based on the medium of H_2_O_2_ is of practical importance in clinical and biological systems. Recently, the interaction between H_2_O_2_ and metal oxides has been applied extensively to the construction of H_2_O_2_-related colorimetric or fluorescent biosensing platform [27,28,29]. Among a wide variety of metal oxides, Fe_3_O_4_ and CeO_2_ were the two best materials for the successful application of the H_2_O_2_-based bioanalysis [27,28,29]. However, the fabrication of H_2_O_2_-based analyses for special targets through the direct action of H_2_O_2_ and metal oxides to modulate the content of applied metal oxide as the signal is still rare.

Recently, MnO_2_ nanosheets, a metal oxide nanomaterial with ultrathin layers, were recognized as planar structures like graphene [30]. Due to their two-dimensional, adsorption and convenient preparation characteristics, several applications based on the changes of the content of MnO_2_ nanosheets, such as fluorescent and drug delivery platforms [31], were established. Besides them, sensing methods for ascorbic acid, hypochlorite and other substances based on the change of the absorbance of MnO_2_ nanosheets, have proved that MnO_2_ nanosheets can be a suitable candidate for the establishment of visual inspection methods [32,33]. Furthermore, MnO_2_ nanosheets have the advantages of non-toxicity and a large molar extinction coefficient (9.6 × 10^3^ M^−1^ cm^−1^) [34,35]. However, colorimetric assays based on the direct application of MnO_2_ nanosheets as the visual signal source is still scarce at present.

**Scheme 1:**
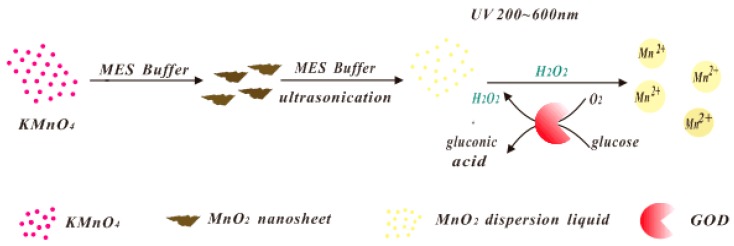
Schematic illustration of the detection for H_2_O_2_ and glucose based on MnO_2_ nanosheets as the visual signals.

In this work, we designed and prepared MnO_2_ nanosheets with obvious yellow color, and used their absorbance and color to fabricate a visual analytical system for glucose monitoring, as shown in Scheme 1. It is known that H_2_O_2_ can react with MnO_2_ nanosheets following the redox reaction of MnO_2_ + H_2_O_2_ + 2H^+^ → Mn^2+^ + 2H_2_O + O_2_. Under certain conditions, the yellow MnO_2_ nanosheets were reduced to colorless Mn^2+^ by H_2_O_2_. The degree of reaction was closely related to the concentration of H_2_O_2_, so we can quantify the concentration of H_2_O_2_ using the color reduction of yellow color of MnO_2_ nanosheets [5]. As the well-known byproduct of the enzymatic reaction of glucose oxidase (GOD) towards glucose, the generated H_2_O_2_ in glucose sensors was the actual target for the modified signal. Therefore, based on the reaction of yellow MnO_2_ nanosheets and H_2_O_2_, the quantification of glucose corresponding to the different absorbance of yellow MnO_2_ nanosheets will be achieved.

## 2. Experimental

### 2.1. Chemicals and Materials

Glucose, 30% H_2_O_2_, and KMnO_4_ were obtained from Sinopharm Chemical Reagent Co., Ltd. (Shanghai, China). 2-Morpholinoethanesulfonic acid (MES), N-ethylmaleimide (NEM), glucose oxidase from *Aspergillus niger* (GOD) (Art. No. G7141) and human serum albumin (HSA) were purchased from Sigma-Aldrich Co. Ltd. (St. Louis, MO, USA). Maltose, fructose, phenylalanine (Phe), lysine (Lys), cysteine (Cys), glutathione (GSH) and sodium periodate were obtained from Aladdin Chemistry Co., Ltd. (Shanghai, China). All chemicals were of analytical grade and used as received. All of the stock solutions were prepared through deionized water.

### 2.2. Apparatus

Ultraviolet and visible (UV-vis) spectra were performed on a UV-2450 spectrophotometer (Shimadzu Co., Kyoto, Japan). FTIR spectra were performed on an Avatar-330 infrared spectrometer (Nicolet Co., Waltham, MA, USA). Transmission electron microscopy (TEM) and high resolution TEM (HRTEM) were performed on a JEM-2100 system (JEOL Co., Ltd., Tokyo, Japan) with an accelerating applied potential of 200 kV. All the testing was carried out at ambient temperature.

### 2.3. Synthesis of MnO_2_ Nanosheets

In a tube, 9 μL of 10 mM KMnO_4_ was added in 75 μL of 0.1 mM MES buffer (pH = 6.0). Afterward, the above solution was diluted to 300 μL with deionized water. After reacting for a few minutes, the color of the solution changed from purple to brown under shaking. The as-formed precipitate was successively centrifuge for 10 min at 3500 g. Then, the precipitate was washed and centrifuged with deionized water several times to remove byproducts and residual ions [36]. Later, the obtained MnO_2_ nanosheets were all dispersed in 100 μL MES buffer in a tube for subsequent use. Such MnO_2_ nanosheets dispersion in a tube was designed as “solution 1”.

### 2.4. Reaction between H_2_O_2_ and MnO_2_ Nanosheets

Before the detection of glucose, the reaction between H_2_O_2_ and MnO_2_ nanosheets was investigated. In 10 mM MES buffer (pH = 6.0), a series of concentration of H_2_O_2_ were respectively added into the solution 1. The reaction volume was adjusted to 700 μL via the addition of 10 mM MES buffer. Then, UV-vis spectrum was recorded.

### 2.5. Determination of Glucose

There are two operating steps for the detection of glucose. First, different concentrations of glucose solution was successively added into the certain volume of 10 mM MES buffer (pH = 6.0) and 57.62 μL of 0.0321 mg/mL GOD, followed by successively mixing and incubation at 37 °C for 5 min to obtain H_2_O_2_. Then, the dispersion of the prepared MnO_2_ nanosheets in a tube (Solution 1) was added into the above solution. The reaction volume was adjusted to 700 μL by controlling the amount of MES buffer. The resulting solution was successively incubated at 37 °C for 40 min for the further UV-vis determination and taking pictures.

## 3. Results and Discussion

### 3.1. Characterization of MnO_2_ Nanosheets

**Figure 1:**
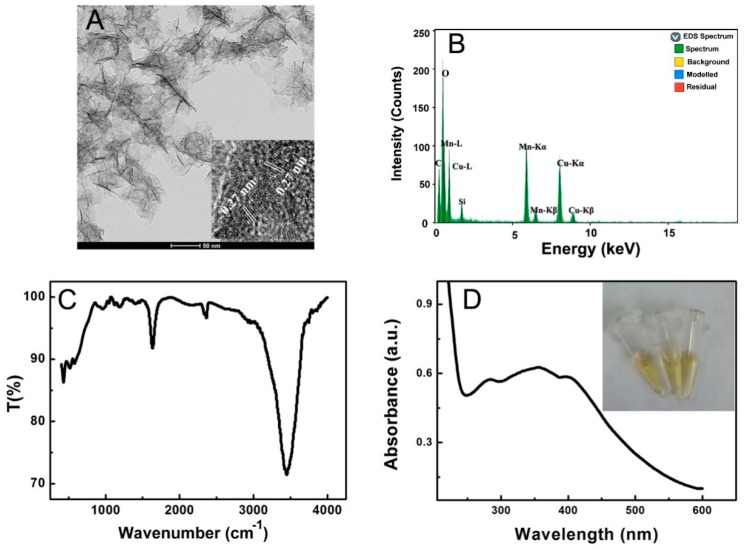
TEM image (**A**), EDS (**B**), FTIR spectrum (**C**) and UV-vis spectrum (**D**) of MnO_2_ nanosheets. Inset of (**A**,**D**) showed the HRTEM of MnO_2_ nanosheets (**A**) and dispersion pictures of MnO_2_ nanosheets of three parallel operations (**D**), respectively.

At first, the morphology, lattice structure and spectral properties of the as-formed MnO_2_ nanosheets were systematically characterized. As displayed in Figure 1A, TEM images of MnO_2_ nanosheets show uniform large two-dimensional film morphology with a lateral diameter of about 100 nm. Furthermore, obvious wrinkles can be seen on the surface of the MnO_2_ nanosheets, suggesting the thin characteristics of the film. High-resolution TEM (HRTEM) demonstrated that MnO_2_ nanosheets formed typical interlayer lattice planes of ~0.27 nm, which was ascribed to the (111) diffraction facet of monoclinic-stuctured δ-MnO_2_ [37]. EDS of MnO_2_ nanosheets in carbon-coated Cu supported membrane suggested the main components of Mn and O with the elemental ratio of Mn and O close to 1:2 (Figure 1B). In addition, FTIR and UV-vis measurements were used for the further illuminating the characteristics of the prepared MnO_2_ nanosheets. As shown in Figure 1C, the FTIR spectrum showed a characteristic absorption peak at about 521 cm^−1^ ascribed to Mn-O vibration, suggesting the specific structure information of the MnO_2_ nanosheets [31,38]. In the wavelength range from 300 nm to 500 nm, the UV-vis spectrum indicated a broad absorption band with a shoulder centered at 346 nm (Figure 1D), which was the d-d transition of Mn (IV) in the lattice structure of MnO_6_ octahedra of MnO_2_ nanosheets. Thus, the absorption at 346 nm can be a suitable selection for the quantification of the content of MnO_2_ nanosheets. The spectral characterizations confirmed the structural information and spectral propertyof the prepared MnO_2_ nanosheets [33]. Moreover, the insert of Figure 1D illustrated that the dispersion MnO_2_ nanosheets exhibited a homogeneous, transparent, and uniform yellow color by comparing three MnO_2_ nanosheet dispersions manufactured in the same manner under parallel operation.

### 3.2. Feasibility of the Detection of Glucose Using MnO_2_ Nanosheets

**Figure 2:**
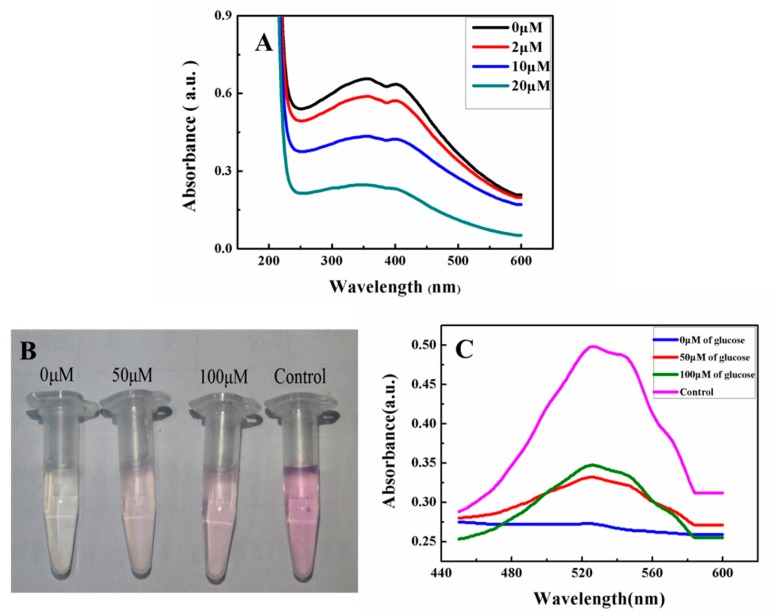
UV-vis spectra of MnO_2_ nanosheets after reacting different concentrations of H_2_O_2_ (**A**). Visualization (**B**) and UV-vis spectra (**C**) of the verification of generatedMn^2+^ from MnO_2_ nanosheets after reacting different concentrations of glucose through GOD using the oxidization by periodate. The control solution shown in (**B**,**C**) was simple KMnO_4_ solution (0.3 mM).

The feasibility of the reaction between MnO_2_ nanosheets and H_2_O_2_ was evaluated. As shown in Figure 2A, with the increased concentrations of H_2_O_2_ in the dispersion of MnO_2_ nanosheets, the absorbance of MnO_2_ nanosheets gradually decreased. The result proved that the redox reaction of MnO_2_ and H_2_O_2_ had happened according to the reaction MnO_2_ + H_2_O_2_ + 2H^+^→Mn^2+^ + 2H_2_O + O_2_. It is known that H_2_O_2_ can be generated under the enzymatic catalysis of GOD to glucose. Thus, the detection of glucose using MnO_2_ nanosheets as signal with the catalysis of GOD can be achieved.

Although the application of MnO_2_ nanosheets as a signal source for the detection of glucose [33], the judgment of the mechanism of the sensing feasibility for glucose is also an important factor for the understanding of this method. It is known that one of the resultant products of MnO_2_ and H_2_O_2_ was Mn^2+^, thus in order to judge the feasibility of the sensing of glucose, the verification of Mn^2+^ as the product of detecting glucose using MnO_2_ and GOD as the reacting sources was investigated. The sodium periodate oxidation colorimetric assay was used to validate the generated Mn^2+^. Mn^2+^ will be oxidized by periodate to generate purple permanganate under acidic conditions following the reaction of 2Mn^2+^ + 5IO_4_^−^ + 3H_2_O → 2MnO_4_^−^ + 5IO_3_^−^ + 6H ^+^. According to the detecting protocol of glucose, the result reacting solutions with different contents of glucose were applied to react with sodium periodate to prove the generation of Mn^2+^. Figure 2B shows that the resulting solution according to the detection protocol of glucose using MnO_2_ nanosheets restored the purple observation with the addition of sodium periodate and the increased concentrations of glucose cause a deepened purple. The increased absorbance was in agreement with the increasing generation of MnO_4_^−^ according to increased Mn^2+^ obtained from the detecting progress of glucose through the reaction of H_2_O_2_ and MnO_2_ nanosheets (Figure 2C). Thus, the feasibility of the reaction between MnO_2_ and H_2_O_2_ can be expanded to the sensing of glucose.

### 3.3. Optimization of Experimental Conditions

**Figure 3:**
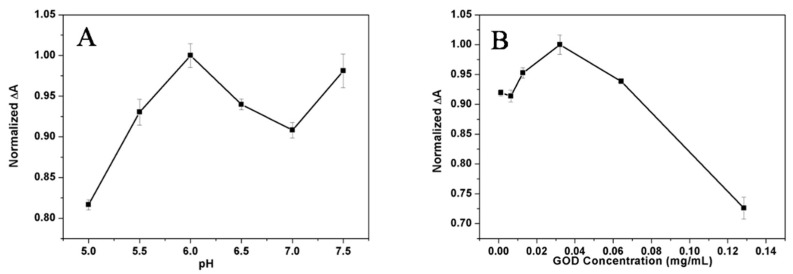
Effects of pH of the assay on detecting 50 μM glucose (**A**), and effects of the concentration of GOD on detecting 0.2 mM glucose (**B**) at 346 nm. Note: Error bars represented the SDs from three independent detections.

pH significantly affects the activity of GOD and the detection sensitivity, so the pH was first optimized. Typically, considering the activity of GOD and the physiological condition of organisms, the main pH range from 5.0 to 7.5 was applied to investigate the influences of MES buffers at different pH on the varied absorbance of the strategy for glucose. Figure 3A shows that the ΔA (the difference of the absorbance of the assay without and with the presence of glucose) of MnO_2_ nanosheets at 346 nm increased and then decreased in the pH range of 5.0 to 7.5 with a maximum value at pH 6.0 towards the detection of glucose. Therefore, pH 6.0 was chosen as the optimal solution for this procedure. A series of the original concentrations of GOD added into the assay condition were also compared to optimize the suitable concentration of GOD for the proposed method, as shown in Figure 3B. The ΔA of MnO_2_ nanosheets at 346 nm was maximum at the condition of the addition of 0.0321 mg/mL GOD into the solution. Thus, original 0.0321 mg/mL GOD for the assay was chosen.

### 3.4. Analytical Performance of the Sensing of Glucose

**Figure 4:**
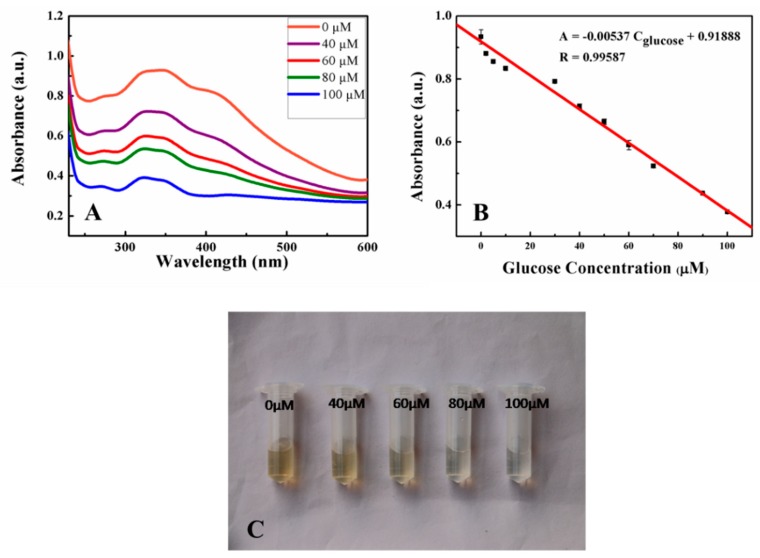
UV-vis spectra of the detection of different concentrations (0, 5, 50, and 100 μM) of glucose (**A**), the linear relationship between the absorbance at 346 nm and glucose concentrations (**B**), and the visual detection of variable glucose (**C**). Error bars in (**B**) represented the SDs from three independent detections.

Under the optimal conditions, different concentrations of glucose were detected. Figure 4A shows the UV-vis spectra after the system of GOD and MnO_2_ nanosheets with several variable concentrations of glucose. The absorbance of the resulting solution decreased proportionately with the increasing concentrations of glucose. Furthermore, Figure 4B indicated that the absorbance at 346 nm showed a linear relation with the glucose concentrations in the range of 0 to 100 μM. The linear fitting equation was A = −0.00537 C_(glucose, μM)_ + 0.91888 with R = 0.9959, and the limit of detection was calculated to be 12.8 μM at a signal-to-noise ratio of 3. It indicated MnO_2_ nanosheets could be utilized as a quantitative and sensitive measuring substrate for glucose detection.

Correspondingly, we could also observe directly the color changes due to different concentrations of glucose. As shown in Figure 4C, the colors of the detection solutions gradually became lighter with the increase of glucose concentration, and the color differentiation of each sample was obvious. It further demonstrated that this method could achieve the colorimetric detection of glucose concentrations.

**Figure 5:**
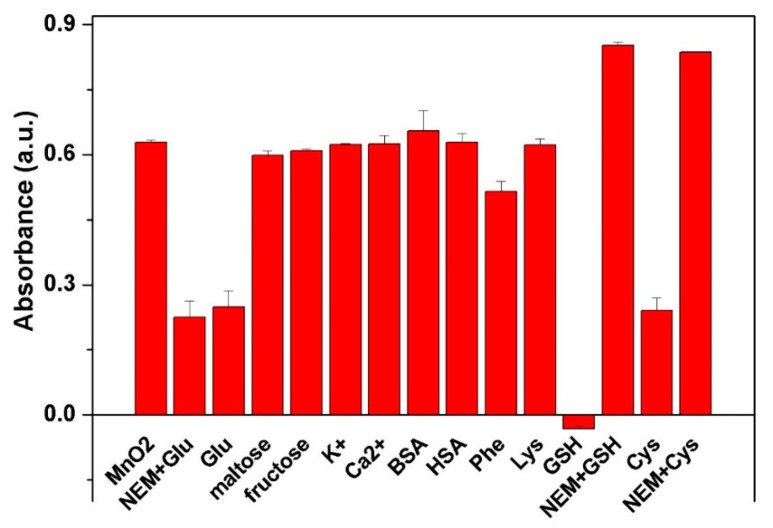
Comparision of the specificity of this method for the detection of 50 μM glucose towards several common interferences of maltose, fructose and metal ions (500 μM), and BSA, HSA (1%) and GSH, Cys (50 μM). Note: Error bars represented the SDs from three independent detections.

Several common interferences were tested to evaluate the specificity of the proposed colorimetric detection. Herein, the dispersion of MnO_2_ nanosheets in the testing condition was diluted to make the absorbance value to be around 0.63, and the UV-vis absorbance of the method toward interference was obtained under the same experimental condition for each other, as shown in Figure 5. Ascribed to their reducibility, GSH and Cys could reduce MnO_2_ nanosheets, resulting in decreasing absorbance. However, when NEM was added, which can selectively consume the thiol group-containing materials, MnO_2_ nanosheets could stabilize by means of resisting the reduction of GSH or Cys. It indicated that NEM could be used to treat the complex samples containing thiol group to eliminate the possible interference. In the system containing maltose, fructose or amino acids, the absorbance of the testing conditions were almost equal to that of pure MnO_2_ nanosheets, indicating that these common interferents would not affect the determination of glucose. Furthermore, the close absorbance for glucose with or without NEM suggested that NEM had negligible effect for the determination of glucose. Thus, the above result suggested the excellent reliability and selectivity of this method for glucose.

### 3.5. Application of Visual Detection in Human Serum Samples

**Figure 6:**
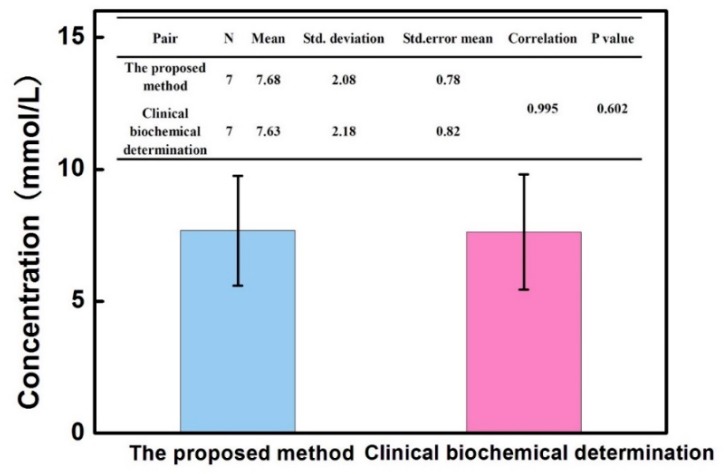
Statistics, correlation and the paired *t*-test of the proposed method and clinical biochemical determination. Error bars showed the varied concentration of glucose in different clinical samples.

The feasibility of the proposed colorimetric assay for possible clinical application was investigated by analyzing seven serum samples obtained from a local hospital. Taking into consideration the blood glucose level in healthy people and the analytical performance of this method, the obtained serum was diluted 100 fold with MES buffer for the detection. NEM was added into the diluted serum to eliminate the possible interfering effect from GSH and other thiol-substances. The collection and use of clinical serums obtained from anonymous participants were approved by the ethics committee of Fujian Medical University. As shown in Table 1, the test results of each sample with low RSDs were obtained using the current method. Compared with the established clinical biochemical determination, the absolute values of the relative errors of each other were lower than 6%. Furthermore, statistical analysis of the detecting results from this method and the clinical biochemical method was operated to prove the real practicability of the proposed method based on MnO_2_ nanosheets. The two methods correlated well with a correlation coefficient of 0.995, as shown in Figure 6; the P-value of the paired t-test was 0.602 (no significant difference). The real application result suggested the excellent accuracy and feasibility of the proposed method based on MnO_2_ nanosheets as simple and effective signals for colorimetric detection of blood glucose.

## 4. Conclusions

In this work, we designed and prepared MnO_2_ nanosheets through a convenient and economical route. After maximizing the UV-vis absorption properties of MnO_2_ nanosheets, we developed an effective method for the visual detection of glucose levels. Furthermore, the proposed assay, which was used to test the clinical serum samples, exhibited accuracy, suggesting the feasibility of the assay in future real applications. In brief, this study suggested an effective strategy for visual signals based on MnO_2_ nanosheets that can be used to assess glucose levels.

## Figures and Tables

**Table 1:** Comparison of test results of the proposed method with clinical biochemical determination.

Samples	Found by the Proposed Method (mmol/L)	RSD (%) (*n* = 3)	Found by Clinical Biochemical Determination (mmol/L)	Relative Errors (%)
1	5.64	8.1	5.52	2.17
2	4.58	4.3	4.52	0.88
3	8.68	2.0	8.99	3.34
4	9.72	1.4	9.98	−2.61
5	9.74	1.4	10.03	−2.19
6	6.91	1.5	6.52	5.37
7	7.87	3.1	7.86	0.13
